# Evaluation of nerve growth factor serum level for early detection of leprosy disability

**DOI:** 10.11604/pamj.2020.37.145.15213

**Published:** 2020-10-13

**Authors:** Dhelya Widasmara, Sri Linuwih Menaldi, Agus Turchan

**Affiliations:** 1 Department of Dermatology and Venereology, Faculty of Medicine, Brawijaya University, Malang 65145, East Java, Indonesia,; 2 Department of Dermatology and Venereology, Faculty of Medicine, University of Indonesia, Depok 16424, West Java, Indonesia,; 3 Department of Neurosurgery, Faculty of Medicine, Airlangga University, Surabaya 60132, East Java, Indonesia

**Keywords:** Detection, disability, early, growth, leprosy, nerve

## Abstract

**Introduction:**

this research aimed to analyze nerve growth factor (NGF) contents as diagnostic tools for early disability in leprosy patients and the cut-off point value.

**Methods:**

research samples consisted of 79 leprosy patients with disability grade 0 or 1 who met the clinically approved inclusion criteria. The age of patients ranged from 14 to 50 years. For both sample groups, blood serum was collected to determine NGF concentration. NGF level was analyzed by enzyme-linked immunosorbent assay (ELISA) according to the manual guide of the kit insert from Cussabio®. Statistical analysis used SPSS 17 software for Windows. A comparison was performed with the Student's t-test and the NGF concentration cut-off point was determined using a receiver operating characteristic (ROC) curve.

**Results:**

the research result demonstrated that NGF concentration in multibacillary leprosy with disability grade 0 was higher than in grade 1. Leprosy with disability grade 0 had an NGF content reaching 100.46 pg/mL, while those with grade 1 had a lower concentration of NGF at 30.56 pg/mL. The higher disability grade indicated a lower NGF concentration in the blood serum. Based on the ROC analysis result, the NGF cut-off was shown to be 81.43 pg/mL. This result indicated that low NGF in nerve and skin lesions of leprosy patients contributes to early peripheral nerve malfunction due to Mycobacterium leprae infection.

**Conclusion:**

these results prove that NGF can be used as a marker of early disability in leprosy, with the cut-off value at 81.43 pg/mL.

## Introduction

Leprosy is a chronic infectious disease caused by *Mycobacterium leprae* infection to the peripheral nerves, which subsequently spreads to the skin and other tissues [1,2]. *M. leprae* has the potential to infect Schwann cells in the peripheral nervous system and causes disorder of sensory, motor, and autonomic nerve functions [3,4]. The malfunction of the peripheral nervous system and neuritis during leprosy reaction causes deformities of patients [5]. The disability level of a leprosy patient is classified as grade 0 (sensibility impairment is not found, and deformity of feet, hands, and eyes cannot be seen) or grade 1 (sensibility disorder appears without deformity of feet and hands or severe visual impairment) [6]. According to the Weekly Epidemiological Report regarding leprosy, Indonesia was ranked third, with 21,026 cases in 2009 [7]. The Indonesian government has established various efforts to avoid disability in leprosy patients, including early detection of infection before deformities occur, medical support, regular assessment of nervous function, rehabilitation, socialization, and personal care. Initial identification of disability has been an obstacle to finding proper disfiguration diagnosis. Nervous cell impairment in leprosy is a result of peripheral nerve cell demyelination. This occurs due to the infection of *M. leprae*, with Schwann cells the main target [8]. Schwann cell demyelination is triggered via activation of the c-Jun route [9]. This consequently inactivates Krox-20, a myelination regular gene. When Schwann cells encounter damage, as an autonomic defense mechanism, they repair the condition via remyelination. This process is affected by NRG1 and NGF as neurotrophic factors, as well as the presence of peripheral myelin protein 22 (PMP22) and protein 0 (P0) since they are basic components of specific myelin in peripheral nerves [10,11].

One strategy to detect early peripheral nerve malfunction due to *M. leprae* infection is recognizing Schwann cell behavior through synthesis marker alteration of the myelin sheath. These markers include NGF, NRG1, Krox-20, P0, and PMP22 [12]. Therefore, this study utilized NGF as a marker for early deformities in leprosy. NGF belongs to the neurotrophin family and has an important role in developing and maintaining cell phenotypes in the peripheral nervous system, also preserving cholinergic nerve integrity in the central nervous system [13]. NGF is endogenously produced during development and maturation by multiple cell types, including neurons, Schwann cells, oligodendrocytes, lymphocytes, mast cells, macrophages, keratinocytes, and fibroblasts. Its primary function in the nervous system is participation in inflammatory processes and immune responses [14]. NGF concentrations are increased during inflammatory tissue processes, producing hyperalgesia through direct activation of nociceptors, which leads to central nervous system activation and neurogenic inflammation [15]. In turn, this process leads to the release of histamine and increased numbers of mast cells and other immune system cells. Inflammatory edema is also important in the neuropathy associated with leprosy, which can induce the degeneration of neural fibers. Strong positive correlations exist between the levels of NGF, NGF-R, and TGF-β in patients with leprosy. This indicates that these factors have synergistic actions that reduce tissue damage resulting from nerve injury [16]. Anand *et al*. (1994) detected low NGF levels in nerve and skin lesions of patients with leprosy and demonstrated that these low NGF levels contributed to the loss of NGF-dependent nociceptive fibers in damaged skin. Schwann cells produce NGF in response to axonal degeneration. However, while the levels of NGF are sharply reduced in the affected nerve trunks of patients with neuropathic lesions, there is a local increase in NGF levels in patients with chronic cutaneous hyperalgesia.

Higher levels of NGF are associated with lepromatous forms, and the increased NGF expression stimulates the expression of TGF-β, which reduces tissue damage resulting from nerve injury. Moreover, NGF restores sensitivity and exerts proliferative and antiapoptotic effects on keratinocytes and endothelial cells [17]. Peripheral nerve injury, or any pathological condition that causes an interruption between the target organ and the nerve cell body, acts as a signal that induces non-neural cell populations (e.g. fibroblasts) to produce NGF. The induction of NGF synthesis in these cells is also modulated by cytokines, which invade the site of nerve injury where nerve regeneration is initiated [18]. Moreover, NGF has been shown to play important roles in affecting injury-specific responses through proinflammatory effects on neutrophils, eosinophils, mast cells, and T lymphocytes [19]. The interactions of NGF in the tissue microenvironment are complex, and its relation to TNF-α, which can induce apoptosis in Schwann cells by binding to specific death receptors, may lead to antagonistic effects. This is because NGF can activate survival signals in the target cell. The same cytokine may have antagonistic effects depending on its interactions with specific receptors, and the intracellular cascade is activated after the activation of these receptors [20]. This supports the assumption that NGF has a crucial role during the Schwann cell myelination process in the peripheral nervous system. Therefore, this research aimed to analyze the validity of NGF concentration as a diagnostic tool for early deformities in leprosy patients and to determine its cut-off point.

## Methods

**Study population:** the sample population consisted of 79 multibacillary (MB) type leprosy patients with disability grades 0 (n = 36) and 1 (n = 43), meeting the clinically approved inclusion criteria and selected by consecutive sampling technique. Their ages ranged from 14 to 50 years, and they were willing to participate in this research. Participants were recruited for five months at the Leprosy Kediri Hospital, East Java, Indonesia. All MB type leprosy patients who met the sample requirements and were willing to participate in the study were given an explanation of the research. Participants gave written informed consent and approval for biopsy of skin lesions. This study was approved by the Health Research Ethics Committee of the Faculty of Medicine, Airlangga University, Surabaya, with information on ethical conduct ("Ethical Clearance") No. 341/EC/KEPK/FKUA/2014. Privacy and confidentiality were supported by principles of the Belmont Report: beneficence, justice, and respect for persons. During the informed consent process, the author informed participants about the precautions taken to protect the confidentiality of the data and the parties who may have access (e.g. research team). This allowed participants to decide about the adequacy of the protections and the acceptability of the possible release of private information to the interested parties.

**NGF measurement:** basic data were recorded for each blood sample from the 79 patients to examine NGF concentration. Both sample groups were examined to quantify the NGF content in blood plasma. ELISA was performed according to the manual guide of the kit insert from Cussabio®. Capturing antibody (human monoclonal anti-NGF) was coated on the ELISA plate in coating buffer, pH 9.8, followed by incubation at 4°C. for 24 hours. It was then washed three times with washing solution (0.15 M NaCl + 0.05% Triton X 100 + 0.02 g NaN_3_in 1 L dH_2_O). Blocking was done with 200 μL of blocking buffer (1% bovine serum albumin + 0.02 g NaN_3_ in phosphate buffer saline, pH 7.0) per well, followed by incubation at room temperature (RT; 20-25°C) for one hour. After washing three times, 100 μL of blood plasma of patients and standard proteins (50, 25, 12.5, 6.25, 3.25, and 1.562 pg/mL) were given per well. Subsequently, the plate was incubated at RT for one hour and washed three times. Horseradish peroxidase-labeled detection antibody (rabbit anti-human NGF; 100 μL) was added per well and incubated at RT for one hour. After washing three times, 50 μL tetramethylbenzidine (TMB) substrate was added following incubation at RT for 30 minutes. This reaction was stopped by adding 1 N H_2_SO_4_. An ELISA reader was employed to determine absorbance with 450 nm wavelength.

**Statistical analysis:** in this study, the distinct values recorded as categorical variables (disability grades 0 and 1) were indicated as percentages, and continuous variables were expressed as mean ± SD based on their normal distribution. Principal-component analysis, followed by a logistic regression model and receiver operating characteristic (ROC) curve analysis, was applied to evaluate the diagnostic potential of the selected NGF level. Before ROC analysis, the results of the study were changed to a continuous scale and returned to the original scale after the cut-off point was found. The cut-off point determination was based on the optimal sensitivity and specificity values on ROC analysis. Statistical analysis used SPSS 17 software for Windows. The results were indicated as adjusted odds ratios (ORs) and 95% confidence intervals (CIs), and all p values were two-tailed, with the significance set at 0.05. Two variables were analyzed using the t-test method to determine any significance in differential expression identified in this study (patients with grade 0 and grade 1), which would suggest a diagnostic potential of disability in leprosy through NGF serum level.

## Results

**NGF concentration:** serum NGF concentrations were significantly higher in disability grade 0 than in disability grade 1 (100.46 ± 48.28 pg/mL vs 30.56 ± 18.95 pg/mL; p < 0.05). The results of the two-tailed t-test showed the value of F = 22.098 with p = 0.000 (p < 0.05) (Table 1). This means that there were significant differences in blood plasma NGF levels between MB leprosy patients with grade 0 and grade 1 disability.

**Table 1:** t-test of NGF concentration level in the blood plasma of MB leprosy patients

Groups	n	Mean (pg/ml)	Deviation Standard
Disability Grade 0	36	100.46	48.28
Disability Grade 1	43	30.56	18.95
F	22.098		
P	0.000		

**Figure 1:**
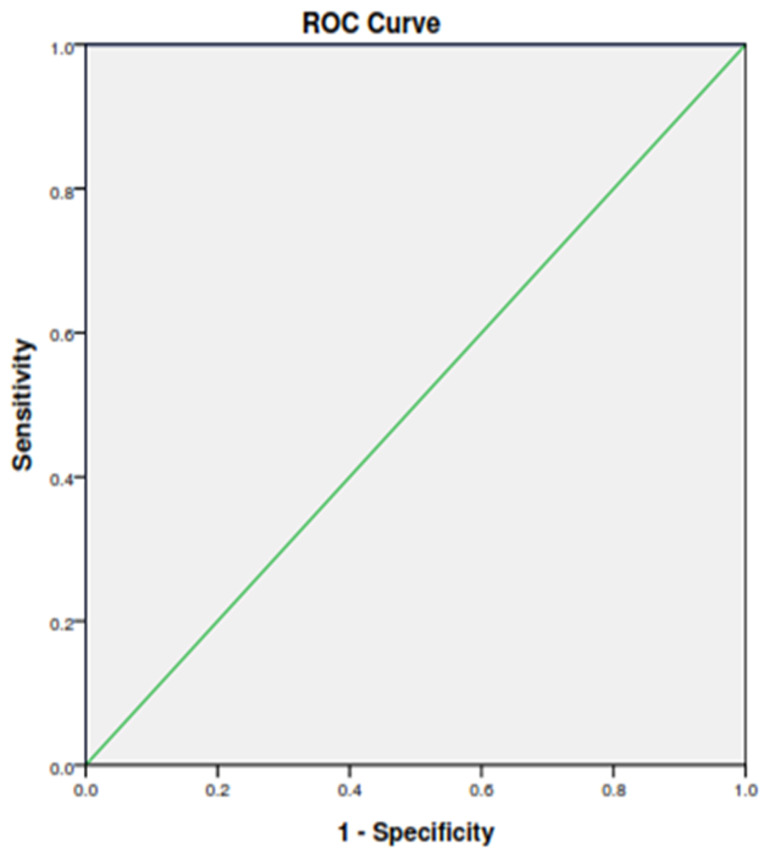
ROC curve based on NGF concentration in samples

**ROC curve analysis of NGF:** according to the ROC curve (Figure 1), the optimal cut-off value of serum NGF concentrations as an indicator for early disability in leprosy was 81.43 ng/L, with a sensitivity of 100% and a specificity of 100% [AUC 0.719; 95% CI (0.719-0.918); p < 0.05] (Table 2). Thus, the reduction of NGF concentration in disability grade 0 was higher than in disability grade 0.

**Table 2:** NGF concentration in leprosy MB plasma

NGF concentration	Sensitivity	1-Specitivity	NGF concentration	Sensitivity	1-Specivity
7.1930	1.000	1.000	37.2000	1.000	0.442
9.5590	1.000	0.977	38.4085	1.000	0.419
11.1250	1.000	0.953	39.3250	1.000	0.395
11.9085	1.000	0.930	40.5245	1.000	0.372
12.7420	1.000	0.884	42.3395	1.000	0.349
13.3245	1.000	0.860	45.0710	1.000	0.326
14.4495	1.000	0.837	47.7560	1.000	0.302
15.2835	1.000	0.814	49.1220	1.000	0.279
15.8565	1.000	0.791	50.5345	1.000	0.256
16.4400	1.000	0.767	53.2660	1.000	0.233
17.8060	1.000	0.744	55.1450	1.000	0.209
19.7640	1.000	0.721	56.5110	1.000	0.186
21.1300	1.000	0.698	58.5530	1.000	0.163
23.0885	1.000	0.674	59.9185	1.000	0.140
24.4545	1.000	0.651	61.4610	1.000	0.116
25.9495	1.000	0.628	63.3110	1.000	0.093
27.7785	1.000	0.605	64.4930	1.000	0.070
29.1445	1.000	0.581	67.2040	1.000	0.047
31.1025	1.000	0.558	72.0670	1.000	0.023
32.2000	1.000	0.535	81.4325	1.000	0.000
32.5105	1.000	0.512	89.4700	0.972	0.000
34.1445	1.000	0.448	91.8250	0.944	0.000
35.8340	1.000	0.465			

## Discussion

Leprosy is a chronic disease caused by *M. leprae*, which infects the skin, peripheral nerves, and other organs of the body, resulting in permanent disability. The strategy in this research to determine impairment of peripheral nerves at the initial stage was by observing NGF concentration. By recognizing its cut-off point value, NGF can be used as a diagnostic tool for early nerve damage. The nerve is an important growth factor to develop and maintain cell phenotypes in the peripheral nervous system and preserve cholinergic nerve integrity in the central nervous system [13]. Another study reported that NGF increased Schwann cell myelination through axonal signal regulation and caused the main axons to be unreceptive to remyelination by oligodendrocytes. Furthermore, that study indicated that NGF displayed an inhibitory function to myelination by oligodendrocytic cells. One of the myelination effects stimulated by NGF was increasing axon diameter, which is closely related to the myelination process. The research showed that besides expanding axon diameter, NGF also increased axonal signals, serving as a support to peripheral nerve myelination. It also found that NGF contributed to reducing myelin in oligodendrocytes [21].

The present study discovered that NGF concentrations in the blood plasma of MB type leprosy patients with disability grades 0 and 1 were significantly different (p = 0.0000). In accordance with previous research, NGF was revealed as a necessary neurotrophin for myelin synthesis, especially in peripheral nerve structure. However, NGF did not function by itself in the establishment of myelin. It required other factors such as axonal signals and TrkA activation. That research also reported that NGF could regulate the myelination process in the opposite effect between Schwann cells and oligodendrocytes through axonal signals. When NGF concentration was increased in the blood, the axonal signal was sent. The axonal signals that control central myelination are likely to be very similar to those that control peripheral myelination. First, it is thought that nearly all primary sensory and lower motor axons maintain the same myelinated or unmyelinated phenotype along their length through the peripheral nervous system and central nervous system. Second, Schwann cells are capable of myelinating central nervous system axons in certain pathological conditions [21]. Another study showed that overexposure to glial-derived neuron growth factor, a growth factor needed for nociceptor development, increased the proportion of axons that underwent myelination in the peripheral nervous system [22]. Other evidence has shown that NGF content in sciatic nerves declined in the second postnatal week, elucidating the potential myelination tardiness on positive TrkA nerve fibers on the spinal marrow [21]. That model indicated peripheral nervous cell myelination was initiated when NGF concentration in sciatic nerves was high, followed by central nervous system myelination after NGF expression in peripheral nerves was decreased. That hypothesis illustrates how Aδ fibers (type III Group A sensory nerve fibers as afferent fibers of nociceptors) can stimulate myelination in both the central and peripheral nervous systems in spite of the opposite effect of NGF on axonal signals [12]. All of these represent that NGF has a vital role during the myelination process in peripheral nerves.

ROC curve analysis was conducted to determine the limit value of NGF concentration for leprosy with disability grade 0 and grade 1. Leprosy with disability grade 0 had NGF content reaching 100.46 pg/mL, while those with grade 1 had a lower concentration of NGF at 30.56 pg/mL. The limit value conducted from the 79 samples at the true positive rate (sensitivity) was plotted with 1-specificity (false positive rate), from a ROC curve value of 7.1930 to 91.8250. In this research, the ROC of the NGF curve could detect early disability of MB leprosy patients with a cut-off of 81.43 pg/mL (Table 2). This research is the first conducted to determine the threshold of serum NGF in leprosy patients. For this reason, we have not been able to compare the findings of other researchers in determining the threshold value of serum NGF in leprosy patients.

## Conclusion

This research suggests that NGF can be used as a detection marker for early disability in leprosy, with the cut-off value of 81.43 pg/mL. If the NGF concentration is over 81.43 pg/mL, it means the nerve regeneration process is still possible. However, if NGF is below 81.43 pg/mL, it means that peripheral nerve damage has already happened.

### What is known about this topic

- NGF has a vital role during the myelination process in peripheral nerves;
- NGF was revealed as a necessary neurotrophin for myelin synthesis, especially in peripheral nerve structure;
- NGF could regulate the myelination process in the opposite effect between Schwann cells and oligodendrocytes through axonal signals.

### What this study adds

- Receiver operating characteristic (ROC) of nerve growth factor (NGF) curve can detect early disability of leprosy multibacillary patients with cut-off 81.43 pg/mL.
